# Consumption

**Published:** 1880-11

**Authors:** 


### CONSUMPTION.

As one person out of six dies of consumptive disease, every man, woman, and child is more or less directly interested in every thing having a practical bearing in reference to a malady which has already carried millions to the grave, and is destined to destroy millions more. In reading the public papers, the impression might be made on the unthinking that consumption was more easily cured than any other human ailment. The confident manner in which it is announced that this, that, and the other remedy is uniformly successful, and that all that is necessary to procure the same, is to forward a three-cent postage-stamp, and the way is open to a prompt, permanent, and radical restoration, does mislead thousands every year. Cod liver oil, naphtha, medicated inhalation, Bourbon whisky; the injection of solutions of nitrate of silver, the ruthless excision of the tonsils, and pectorals and syrups and troches and a multitude of other remedies, safe, sure, and infallible, have been proposed from time to time, have had their fashion and their butterfly hour; but the people still continue to die of the dreaded disease; not as before, but more numerously than ever, proving beyond contradiction, that there is no curative power in any of them. And the very avidity with which any new remedy is seized upon and published to the world, is evidence enough, that the great want is still unsupplied. When it is remembered that consumption is a general destruction of the substance of the lungs, it ought to be felt, even by the unreflecting, that in reality, there can be no absolute cure: because, when a portion of the lung is once destroyed, its reproduction is as impossible as that of a lost limb or finger. In a literal sense, then, consumption is absolutely incurable. At the same time a man may, from various causes, lose a part of his lungs, and yet have that decay arrested, and live in reasonable health for many years afterward. Anatomists say that in examining the lungs of those who have died after the age of forty-five, it is a very common thing to notice evidences of a partial destruction of the lungs, and their subsequent healing up, without the subjects of this process ever having had a suspicion in life, that any thing was the matter with the lungs. It follows then, that being cured of consumption, in this restricted sense, is an event of every day occurrence. But such a conclusion brings with it very little comfort when connected with that other observation, that such "cures" are always spontaneous, and are never clearly traceable to any drug swallowed, to any gas or atmosphere inhaled, or to any surgical operation. The reputation which successive vaunted remedies have obtained, has been owing to several causes, each of which is particularly calculated to foster a deception; and

First. Consumption is a disease which, in its nature, is of a very flattering character, in that it generally, except in its very last stages, is not attended with any pain; the appetite is good. and the intellect clear; the malady itself is in the lungs, which, being scantily supplied with nerves, have very little feeling.

Second. The seeds of consumption, as previously explained, are little, hard, roundish substances, called "tubercles," scattered through the lungs in little patches, more or less extensive, which patches ripen, as it were, at different times, as apples on a tree or berries on a bush; this ripening, however, is rather a rottening process; it is the softening of the tubercles, whereby the lungs become disorganized and destroyed, and are in this state, spit out of the mouth, in the shape of a thick, yellowish matter. While this softening process is going on, and until the matter is wholly expectorated, the patient does not "feel so well;" there is fever, and there is cough, with a variety of other discomforts. But when the matter of that "patch" is all expectorated, and the lungs are relieved of the discomfort of its presence; the elasticity of the system returns; the cough greatly abates, and in some cases disappears almost entirely, and the patient expresses himself as feeling "almost as well as I ever did in my life." This better feeling continues until another patch ripens, rots, and is spit away; the process going on, in repetitions, from time to time, until such a large portion of the lungs has been destroyed, that enough is not left to live upon, and death closes the scene. Hence it is, that the history of almost every consumptive, is that of being better or worse, through the whole course of its progress; which averages about two years. These "spells" of being "worse" are uniformly attributed to having "taken a little cold." The patient, too willing to be deceived, takes comfort in the reflection, that if he had not taken that last cold, he would still have gotten better; and summoning up a new resolution and energy, determines that he will be more careful against taking cold another time; and as a means of so doing, "bundles up" more; is more guarded as to "exposures:" that is, goes out less, hugs the stove more, leaves less frequently his cozy corner at the fire; not taking note of the fact for a long time that he "takes cold," as he calls it, in spite of all his efforts, and finally settles down in the declaration that the "least thing in the world gives me a cold;" or there is a positive inability to determine how he got his last cold; and then begins to think that it came on of itself; the true state of the case being that it is simply the natural progress of the disease; that "taking cold" had nothing to do with these repeated back sets. And there is a failure also to observe, that during this "bundling up," this fearfulness of "exposures," involving closer and closer confinement to the house, the "colds" come more frequently, last longer and longer, until one runs into another, and there is a continued cold; which means in reality, that the destructive process is now going on steadily, and with it there is a more and more harassing cough, a greater and greater thinning of flesh, a more and more distressing shortness of breath, more drenching night-sweats, more consuming fevers, with a weakness, approaching the utter helplessness of a new-born child.

But suppose in the earlier stages of the malady, when perhaps the first or second or third "patch" had pretty much softened and the patient was beginning to spit it away, a particular remedy was administered; the improvement which always follows the riddance of the yellow matter, which is really rotted lungs, is attributed to the last thing taken or done; it may be a week, a month, or a year before another "patch" of tubercles begins to soften; meanwhile, considerable health is enjoyed and the patient, quite willing to believe that he has been cured of consumption, speaks of the remedy used, in the most extravagant terms; and with a kind of gratitude gives his "certificate" of its value in his own case; and in a month it has been read by millions. Hence the multitude of fallacious "cures," so called, which flood the country.

But suppose there had been but a single "patch of tubercles," and nothing had been done; but there was the usual cough, expectoration, night-sweats, etc., and then an ultimate restoration to health, the whole thing is dismissed with the remark, that it was only a very bad cold.

There is, perhaps, not a man living who is troubled with "a very bad cough," who has not been advised to try a multitude of remedies, with two stereotype statements, "It can do you no harm, if it does you no good;" and "It cured a much worse case than yours."

But there are literally millions who, after hopefully trying the remedy, have been doomed to the sad experience and admission, that "however much others may have been benefited, no benefit has resulted in my case."

But as there are persons who have labored under the more common and unequivocal symptoms of consumption, such as cough, spitting blood, expectoration of yellow matter, night sweats and swollen ankles, and yet have recovered and lived in good health a quarter of a century afterward, it will be instructive to note, what are the circumstances in common, in all these well-authenticated cases; then we may conclude, that if in any given case these circumstances can be brought about, similar favorable and triumphant results may be reasonably anticipated. Let the reader turn to the cases of apparent cure frequently noticed in the pages of this "Journal," and mark the symptoms of each case as there given. To these may be added the case of General Andrew Jackson. It was stated in the public prints at the time of his death, that there was every indication that one fourth of the lungs had been destroyed by disease twenty-five years before. To these may be added a case which came under the author's notice five years ago.

This patient was an Englishman; tall, slim, nervous temperament, a traveling clock-mender and tinker. The yellow matter in the air-passages was so abundant that he could bring up a mouthful at any time, with a kind of gulp, or hem. This seemed to be a case so utterly hopeless in all its aspects, and one wherein no medicine whatever seemed to be appropriate, the only advice which was at the same time applicable and possible to him, (as he was extremely poor,) was that he should eat regularly and as much as possible, and spend his waking existence in some very active exercise out of doors. He was advised to cough as little as possible, to make every effort to repress it, to endeavor to get rid of the "phlegm" by hemming; but that whenever it was not possible to restrain a cough, to throw the head back and cough out at an angle of about forty-five degrees, so as to jar and strain the lungs as little as possible, and thus bring away the phlegm more easily, as would be the case when it came up much nearer in a straight line, than at a right angle, as in ordinary coughing, or at a more acute angle still, when the chin is bent down as it usually is, in the act of coughing. This man was so very poor, (and the winter was approaching,) that it was considered necessary to furnish him with some clothing. But he was well informed; had seen and thought for himself as to the nature and philosophy of his malady; so that there was a sufficient inducement to explain to him the reasons for the particular courses advised; these he seemed to comprehend and appropriate. Still, there was no expectation of ever seeing him again in life. A year later he was heard from through a third person, who spoke of him as the "crazy carrier." Adopting the suggestions made to him, he at once procured the situation of meeting the express railroad train at a certain point, receiving the daily newspapers, which had to be carried on foot to a post-office two or three miles distant. At first he was too weak to walk fast; but by great patience he had increased his gait, until at the time of his reporting, he was literally running five miles every twenty-four hours; never missing a day. On one occasion, when the thermometer was hovering about zero, he was seen without gloves, or overcoat, his hat thrown back, so as to expose the whole forehead, papers under arm, and at a long, loping gait, "making time" for the post-office; this furnished the occasion for giving the sobriquet of "the crazy carrier." Later on he came to pay a fee for the first consultation, and five years from the first interview, he called to say that he was well; that he had supported his old father and mother during the interval; that he was drafted, and wanted to know what could be done for him in the way of securing an exemption. On a careful examination, there was found no physical ground for excusing him from serving as a soldier; and all that could be conscientiously done for him, was to give a certificate that he had been under treatment within a few years for consumptive disease.

In all the cases of apparent restoration from consumptive symptoms above referred to, there is one element, always present, never absent; it is no pill or potion, no drug or "simple" remedy; no syrup nor "pectoral;" no lozenge, no surgeon's operation, nothing physical; but something as impalpable as thin air; it is simply force of will; an unconquerable determination to live above disease; to conquer it or to die in the attempt. Moral courage, then, is at the very foundation of all effective treatment for consumption of the lungs; and is worth a thousand times more than any "dose" ever compounded by the apothecary; or than any "operation," which the most skillful surgeon in existence can boast of. Without this quality of the mind, invincible determination, all artificial means for the cure of consumption in its ordinary course, have seemed to be utterly unavailing. It must not be that fitful bravery which leads a man, in the excitement of the moment, to march up to the cannon's mouth, at the instant of its belching forth flame and fire and death; but it must be a persistent resolution, a "chronic" courage, which remains at the highest point all the time; day in and day out; reaching through days and weeks and months, and even years if need be. A courage which can at a moment's warning leave the cozy fireside and brave the fiercest winds of winter, which can any day abandon the comforts and happiness of home, and undertake long journeys on horseback or foot, through snow and frost and freezing rains; sleeping in comfortless cabins, or by the wayside; living on the coarsest fare of "squatter" poverty, or depending on the precarious "bringing down" of the hunter's rifle; the men who can do these things, and do them with such a will as to make them as mere pastimes, these are the men who can, and who often do, survive for long years, the fierce attacks of consumptive disease, and all honor be to them, for such high types of heroism!

But in this connection let it be borne in mind that such moral courage, such force of character, is not found oftener than once in a thousand, and that this being so, the man who has actual consumption may consider himself inevitably doomed, except in the very rarest number of cases; and that they are wisest who make a systematic effort to live in such a way, as not to fall into the grasp of so remorseless a disease themselves; and to do all that is possible, by judicious counsel and unceasing watchfulness, to preserve their children, and others who may be under them, from those habits of life which invite so fell a malady.

Climate for Consumptives.—It has been a fashion of many years' standing, to go to the South, when the lungs seemed to be affected; or to take long journeys by sea. Of late years, another "notion" seems to have taken hold of the public mind, to wit, that Minnesota, the great North-West, is best adapted toward recovering a man from consumption; and now, the stream of consumptive travelers is in that direction, instead of toward the sunny South, to Cuba, Madeira, and other localities. This change of sentiment originated in loose newspaper statements, that very few persons were noticed to have died of consumption in Minnesota. Similar statements have been made as to California, and for the very same reasons; both countries are comparatively new; few, other than the hardy, "settled" in them, and for obvious reasons the statistics on the subject must have been very imperfectly gathered. California is in a measure inaccessible, by reason of its distance. Havana and other warmer latitudes require more means than the multitude can command; hence, the great army moves toward the "North-West," with most discouraging results. The ablest resident physician at St. Paul, the chief town of Minnesota, says, that two thirds of the consumptives who reach that point, die there; and it is his frank and honorable habit to advise visitors to leave there as soon as possible. The air is indeed pure, and still and dry, having a uniform temperature in mid-winter; but whether from its great severity, or its rarefied character, or from its possessing some stranger ingredient, not yet detected, or whether from other causes, the fact remains the same, that two thirds of all who go to Minnesota for the removal of consumptive symptoms, perish there; and how many, soon after their return to their own homes, there are no means for ascertaining. But it is suggestive to note in the cases given in the preceding pages, that they were from all latitudes, from Canada to Cuba, leaving us to fall back on the great comprehensive fact, that the essential, the fundamental, the all-controlling agency in the arrest of any case of consumptive disease, and a return to reasonable health for any considerable time, is an active, courageous, and hopeful out-door life, in all weathers and in any latitude, with some rousing motive, other than regaining the health, beckoning them on, to do and to dare.

